# *wecB* Gene of *Salmonella* Gallinarum Plays a Critical Role in Systemic Infection of Fowl Typhoid

**DOI:** 10.3389/fmicb.2022.880932

**Published:** 2022-05-26

**Authors:** Shinjiro Ojima, Hisaya K. Ono, Ryo Okimoto, Xiaoying Yu, Makoto Sugiyama, Kazuki Yoshioka, Takeshi Haneda, Masashi Okamura, Dong-Liang Hu

**Affiliations:** ^1^Laboratory of Zoonoses, Kitasato University School of Veterinary Medicine, Towada, Japan; ^2^College of Animal Science, Jilin Agricultural University, Changchun, China; ^3^Laboratory of Veterinary Anatomy, Kitasato University School of Veterinary Medicine, Towada, Japan; ^4^Laboratory of Microbiology, Kitasato University School of Pharmacy, Tokyo, Japan; ^5^Section of Applied Veterinary Sciences, Division of Veterinary Sciences, Department of Veterinary Medicine, Obihiro University of Agriculture and Veterinary Medicine, Obihiro, Japan

**Keywords:** *Salmonella* Gallinarum, fowl typhoid, chicken, *wecB* gene, enterobacter common antigen

## Abstract

*Salmonella enterica* serovar Gallinarum (*S*. Gallinarum) is a host-specific pathogen causing fowl typhoid, a severe systemic infection in poultry, which leads to substantial economic losses due to high morbidity and mortality in many developing countries. However, less is known about the pathogenic characteristics and mechanism of *S*. Gallinarum-induced systemic infection in chickens. In this study, we deleted the *S*. Gallinarum UDP-*N*-acetylglucosamine-1-phosphate transferase gene, which contributes to the biosynthesis of enterobacterial common antigen (ECA), and studied the pathogenicity of this *wecB*::Cm strain in a chicken model of systemic infection. The *wecB*::Cm mutant strain showed comparable growth but lower resistance to bile acid and nalidixic acid than the wild-type strain *in vitro*. In the oral infection model of chickens, the virulence of the *wecB*::Cm strain was significantly attenuated *in vivo*. Chickens infected with wild-type strain showed typical clinical signs and pathological changes of fowl typhoid and died between 6 and 9 days post-infection, and the bacteria rapidly disseminated to systemic organs and increased in the livers and spleens. In contrast, the *wecB*::Cm mutant strain did not cause chicken death, there were no significant clinical changes, and the bacterial numbers in the liver and spleen of the chickens were significantly lower than those of the chickens infected with the wild-type strain. In addition, the expression of interleukin (IL)-1β, tumor necrosis factor (TNF)-α, and CXCLi1 in the livers of *wecB*::Cm-infected chickens was significantly lower than that of the chickens infected with the wild-type strain. Furthermore, the attenuated *wecB*::Cm strain could persistently colonize the liver and spleen at low levels for up to 25 days post-infection and could induce a protective immune response in the chickens. These results indicate that the *wecB* gene is an important virulence factor of *S*. Gallinarum in the chicken model of systemic infection, and the avirulent *wecB*::Cm mutant could possibly be used as a live-attenuated vaccine strain for controlling fowl typhoid.

## Introduction

*Salmonella enterica* serovar Gallinarum biovar Gallinarum (*S*. Gallinarum), a host-specific *Salmonella*, is an important pathogen causing fowl typhoid, a severe systemic infection in poultry, which leads to high morbidity and mortality in chickens, and represents a significant burden for the chicken industry and high substantial economic losses in many developing countries (Shivaprasad and Barrow, 2008; Barrow and Freitas Neto, 2011; Kim et al., 2019). *S*. Gallinarum produces a severe, septicaemic, systemic, and often fatal infectious disease in many kinds of avian birds, especially in chickens (Shivaprasad, 2000; Mdegela et al., 2002). To promote the effective reproduction and growth of chickens and their global trade, attempts have been made to vaccinate chickens with live-attenuated strains of *S*. Gallinarum to control fowl typhoid (Kwon and Cho, 2011; Penha Filho et al., 2016; Wigley, 2017). However, live-attenuated vaccines retain some virulence and the protective effects of the vaccines are not yet completely satisfactory (Penha Filho et al., 2010; Ji et al., 2021). Therefore, investigating the pathogenic factors of *S*. Gallinarum and a better understanding of its systemic infection mechanism are important and necessary for the development of more effective and safer vaccines.

*Salmonella enterica* serovars are a diverse group of gastrointestinal pathogens, including *S*. Enteritidis that has evolved to survive in a wide range of environments and across multiple hosts, *S*. Typhimurium that is a host-specific to mice, and *S*. Gallinarum that has a limited host range and is usually associated with poultry and bird host species, especially chickens (Huang et al., 2019; Kim et al., 2019). *Salmonella* has the enterobacterial common antigen (ECA), which is located in the outer leaflet of the outer membrane and in the periplasm that allows enterobacteria to withstand stress brought about by environmental factors, including heat, pH, salinity, osmotic activity, and antibiotics stresses (Ramos-Morales et al., 2003; Liu et al., 2020). Studies on ECA functions have shown that ECA plays a vital role in bacterial physiology and interaction with the environment in *S*. Typhimurium and *Escherichia coli*, and the awareness of the importance of ECA in bacterial survival and pathogenicity is also increasing (Gilbreath et al., 2012; Mitchell et al., 2018; Rai et al., 2021). The synthesis of ECA is intricate, and the genes necessary for many steps in the synthesis are chromosomally encoded in the *wec* gene cluster, including *wecA* through *wecG* genes (Morgan et al., 1997; Campbell et al., 2000; Bohm et al., 2018; Mitchell et al., 2018). The first step of synthesis involves the formation of *N*-acetyl-D-glucosamine (GlcNAc)-pyrophosphoryl-undecaprenol that uses UDP-GlcNAc as a substrate to attach GlcNAc-1-phosphate to Und-P and is catalyzed by WecA (Rick et al., 1985; Barr and Rick, 1987). *wecB* (a UDP-*N*-acetylglucosamine 2-epimerase) reversibly epimerizes at carbon position 2 between UDP-GlcNAc and UDP-*N*-acetylmannosamine (Sala et al., 1996; Morgan et al., 1997). WecC oxidizes UDP-*N*-acetylmannosamine in the presence of NAD+ to form UDP-*N*-acetyl-D-mannosaminuronic acid (ManNAcA) (Kawamura et al., 1979). The UDP-ManNAcA is the substrate to attach ManNAcA to the lipid IECA carried out by WecG (Barr and Rick, 1987; Barr et al., 1988). This process results in ManNAcA-GlcNAc-pyrophosphoryl-undecaprenol. The complete ECA structure is important to the physiology and pathogenicity of enteric pathogenic bacteria (Barua et al., 2002; Mitchell et al., 2018; Rai and Mitchell, 2020). Previous studies using *S*. Typhimurium strains with defined mutations have reported that the virulence of the ECA mutant is attenuated in mice (Rick et al., 1998; Gilbreath et al., 2012). Ramos-Morales et al. (Ramos-Morales et al., 2003) described a role of two ECA-specific loci (i.e., *wecA* and *wecD*) in bile resistance as well as virulence in animal infections. Random-transposon mutagenesis experiments performed in *S. enterica* revealed that disruption of six ECA operon genes (i.e., *wecB, wecC, wecD, wecE, wecG*, and *wzxE*) led to increased speed of lysis by bacteriophage (Bohm et al., 2018; Rai and Mitchell, 2020). These data suggest that ECA may play a broad role in bacterial virulence and could be important for *Salmonella* pathogenesis in diarrhea, gastroenteritis, and typhoid-like diseases in mammals.

Despite the biochemical characteristics of ECA in a wide range of host *Enterobacteriaceae* and the roles in gastroenteritis and typhoid-like diseases in mammals have been well studied, less is known about the biological function and the pathogenic role of ECA in a host-specific *S. enterica* serovar, *S*. Gallimarum, which causes systemic infection in poultry. In this study, to shed some light on the pathogenic mechanism of *S*. Gallinarum systemic infection in chicken, we constructed an ECA mutant strain (*wecB*::Cm) of *S*. Gallinarum and studied the biological function of ECA using our recently established model of systemic infection in chicken (Ojima et al., 2021). Our results demonstrate, for the first time, that disruption of the *wecB* locus can lead to bile salt and nalidixic acid sensitivity, and the resulting strains are significantly attenuated in the infected chickens *in vivo*. Interestingly, the attenuated *wecB*::Cm strain was not eliminated immediately but rather established a persistent colonization in the chicken without pathogenicity and induced a significant preventive immune response, indicating the possibility of using a *wecB* mutant of *S*. Gallinarum as a live-attenuated vaccine strain for controlling fowl typhoid.

## Materials and Methods

### Bacterial Strains and Growth Conditions

*Salmonella enterica* serovar Gallinarum (*S*. Gallinarum) 287/91, a spontaneous nalidixic acid-resistant strain (Ojima et al., 2021), was maintained in Luria-Bertani (LB) broth plus 30% glycerol at −80°C. Bacteria were routinely grown in LB broth (Eiken Chemical, Tokyo, Japan) at 37°C with shaking (at 150 rpm). For infection experiments in chickens, *S*. Gallinarum 287/91 and a deletion mutant (*wecB*::Cm) were cultured at 37°C in LB broth to logarithmic phase and then collected by centrifugation and washed twice with sterile 0.01 M phosphate-buffered saline (PBS). The washed bacteria were diluted with PBS, adjusted spectrophotometrically at 600 nm to reach 1.0 × 10^9^ colony-forming unit (CFU)/ml, and then were diluted to 1.0 × 10^8^ CFU/ml or 2.0 × 10^7^ CFU/ml. Chloramphenicol (Cm; 20 μg/ml) or ampicillin (Amp; 100 μg/ml) was added to the media when needed.

### Construction of *wecB*::Cm Mutant Strain

*S*. Gallinarum ECA mutant strain (*wecB*::Cm) was constructed in *S*. Gallinarum 287/91 wild-type background using the Lambda Red recombination method (Datsenko and Wanner, 2000). PCR primers, which were 60 bases long, SG3523-F1 (AGGGGGCTGGGCCCCTACTGTCTAT TCGAAGAGAATCGATGTGTAGGCTGGAGCTGCTTC), and SG3523-R1 (TTTCGTCGT GCAGCAGACGCATAACTTCCGCCACAATCCGCATATGAATATCCTCCTTAG), were designed with 40 bp of the 5′ ends corresponding to the ends of the desired deletion and the 20 bp of 3′ ends to amplify the Cm cassettes from plasmid pKD3 (GenBank accession number: AY048742.1). PCR was performed in a 50 μl reaction mix containing 5 μl of 10 × PCR buffer for KOD plus version 2, 5 μl of 2 mM dNTPs, 3 μl of 25 mM MgSO_4_, 3 μl of primer mix (0.3 μM final concentration of each primer), 1 μl of KOD plus version 2 polymerase (1 unit, Toyobo, Osaka, Japan), 1 μl of DNA template (about 0.1 ng pKD3 plasmid), and distilled water. The thermal cycling conditions were initial denaturation at 94°C for 2 min, followed by 30 three-step cycles of denaturation at 98°C for 10 s, annealing at 55°C for 30 s, extension at 68°C for 1 min, and a final extension cycle at 68°C for 3 min. PCR products were electroporated into *S*. Gallinarum carrying pKD46 grown at 30°C in LB broth containing Amp (100 μg/ml) and L-arabinose (10 mM; Sigma-Aldrich, St. Louis, MO, USA). Electroporated bacteria were selected on LB agar plates containing Cm (20 μg/ml) at 37°C. The colonies collected from the LB agar plates containing Cm were streaked onto an LB agar plate and incubated at 42°C to remove thermosensitive plasmid pKD46. The recovered colonies were checked for sensitivity to Amp, and the mutation was confirmed by PCR amplification using a flanking region primer, SG3523-F2 (ATCACGCGGTCATTTTTAAT), and a priming site within the Cm cassette of pKD3, C1 (TTTTCACCATGGGCAAATAT).

### Assays of Growth Kinetics and Sensitivity of *wecB*::Cm Mutant in Various Culture Conditions *in vitro*

Bacterial growth characteristics of *wecB*::Cm mutant were monitored and compared with wild-type strain by measuring the optical density (OD_600 nm_) of bacterial cultures at 37°C with shaking (150 rpm) at 2 h intervals. At the indicated time points, 100 μl of each culture was serially diluted in LB broth and 100 μl of each dilution was spread on an LB agar plate. After overnight incubation at 37°C, colonies on the plates were counted as CFUs. For the resistance assays, bacteria were grown in LB broth for 14 h and diluted to 2.0 × 10^7^ CFU/ml, and 50 μl diluted culture was mixed with 50 μl of LB broth that contained various concentrations of bile acid (Sigma-Aldrich; final concentration was 0.01 or 0.02 mM), hydrogen peroxide (H_2_O_2_) (Wako, Osaka, Japan; final concentration was 0.5 or 1.0 mM), or nalidixic acid (Wako; final concentration was 1.25 or 2.5 mg/ml) in 96-well flat-bottom plates (Greiner Bio-One, Kremsmünster, Austria). The plates were incubated at 37°C without shaking. Bacterial growth at the indicated time points was determined by monitoring the optical density (OD_595 nm_) of bacterial cultures using a 96-well plate reader (Bio-Rad, Model 680 microplate reader, Hercules, CA, USA).

### Chickens and Experimental Infection

All animal experiments were conducted in accordance with the animal experiment rules set out in the Animal Welfare Law and Guide for the Care and Use of Laboratory Animals. All efforts were made to minimize the suffering of the animals during the experiments. Female Boris Brown chickens, which are well known to be susceptible to salmonellosis (Smith, 1956; Ojima et al., 2021), were housed and provided water and food *ad libitum*. To ensure the chickens were free from *Salmonella*, fecal swabs were taken from the transport box for the bacteriological detection of *Salmonella* before experimental infection. For oral infection, each chicken was inoculated by oral gavage either with 10^8^ CFU of wild-type or *wecB*::Cm mutant strain in a volume of 1.0 ml at 20 days old. After inoculation, chickens were reared for 14 days and observed twice a day to monitor their clinical signs. Animal experimentation protocol was approved by the President of Kitasato University through the judgment of the Institutional Animal Care and Use Committee of Kitasato University (Approval No. 20-055 and 21-039).

### Isolation and Enumeration of *Salmonella* in Systemic Sites

For the detection of bacterial counts in the systemic sites of the chickens post-infection, chickens were inoculated by oral gavage with 10^8^ CFU of wild-type or *wecB*::Cm mutant, as described earlier, and were euthanized on 1, 3, 5, and 7 days post-infection. The chickens orally inoculated with 1.0 ml of PBS were used as negative controls. Three to six chickens in each group were euthanized at each time point. The liver and spleen samples were collected aseptically and then homogenized in 9 volumes of PBS. The homogenates were further serially diluted 10-fold with PBS and spread on LB agar plates. After incubating at 37°C for 24 h, the number of colonies on the plate was counted, and it was calculated as a CFU/g organ.

### Clinical Evaluation and Histopathological Examination

The clinical changes in chickens infected with the wild-type or *wecB*::Cm mutant were observed and evaluated for the onset of systemic infection. Clinical signs, redness, and discoloration of the comb and ruffled feathers were observed and recorded. Three to six chickens in each group were euthanized at 1, 3, 5, and 7 days after infection and were investigated for the extent of inflammation by observing redness, swelling, congestion, bleeding, and discoloration of the tissues. To estimate histological changes and inflammation levels, the liver and spleen of each group were fixed in 4% paraformaldehyde (pH 7.4) for 24 h at 4°C and embedded in paraffin wax. Sections were cut at three levels to a thickness of 4 μm and stained by the Hematoxylin-eosin (HE) staining. Histological changes, such as infiltration of inflammatory cells and tissue damages, were recorded for each section.

### Quantitative Real-Time RT-PCR Analysis

To analyze the host responses in the organs of the chickens infected with the wild-type or *wecB*::Cm mutant, five or six chickens in each group were euthanized at 1, 3, 5, and 7 days post-infection, as described earlier. Tissue samples of the liver and spleen were immersed separately in 0.5 ml of RNA*later* (Thermo Fisher Scientific, Waltham, MA, USA) and stored at −80°C until use. Total RNA was extracted from 5 × 5 mm of the tissue using RNAiso Plus (TaKaRa, Kusatsu, Japan) according to the manufacturer's instructions. The quantity and quality of RNA were determined by spectral analysis (NanoDrop 2000, Thermo Fisher Scientific). After being treated with DNase, RNA was transcribed to complementary DNA (cDNA) using the ReverTra Ace^®^ qPCR RT Master Mix (Toyobo), following the manufacturer's instructions. The expression of mRNA for interleukin (IL)-1β, IL-6, tumor necrosis factor (TNF)-α, interferon (IFN)-γ, IL-12, and CXCLi1 in the tissues was measured using quantitative real-time RT-PCR. Primer sequences are listed in Table 1. Notably, 20 μl reaction mixture, which contained 2.0 μl cDNA, 10 μl THUNDERBIRD^®^ SYBR^®^ qPCR Mix, 0.6 μl of each primer (at 10 μM), 0.4 μl 50 × ROX reference dye, and 6.4 μl nuclease-free water, was prepared using the THUNDERBIRD^®^ SYBR^®^ qPCR Mix (Toyobo). Quantitative real-time RT-PCR was performed on StepOnePlus Real-Time PCR System (Applied Biosystems, Foster City, CA, USA) with the following reaction profile: one cycle at 95°C for 20 s, and 40 cycles at 95°C for 3 s and 60°C for 30 s. The melt-curve mode was used to check the specificities of amplified products (one cycle at 95°C for 15 s, 60°C for 1 min, and 95°C for 15 s) after amplification. The expression of the target genes was determined using the cycle threshold value relative to that of the housekeeping gene GAPDH. The results were expressed as fold changes in corrected target gene expression in the infected chickens relative to the uninfected controls.

**Table 1:** Primers for PCR and sequences.

**Primer**		**Sequences (5^′^-3^′^)**	**Amplicon size (bp)**	**GenBank accession no**.
GAPDH	Forward	GGCACTGTCAAGGCTGAGAA	99	NM_204305.2
GAPDH	Reverse	TGCATCTGCCCATTTGATGT		
IL-1β	Forward	CGAGGAGCAGGGACTTTGC	71	NM_204524.2
IL-1β	Reverse	GAAGGTGACGGGCTCAAAAA		
IL-6	Forward	CCTGGCGGCCACGAT	61	NM_204628.2
IL-6	Reverse	CGAGTCTGGGATGACCACTTC		
TNF-α	Forward	GAGGCAGGGAGAAAAATAGGTTTC	83	NM_001037837.2
TNF-α	Reverse	GCTTTTACTATGGGGTAACCAACTC		
IFN-γ	Forward	ATGTAGCTGACGGTGGACCT	102	NM_205149.2
IFN-γ	Reverse	CCAAGTACATCGAAACAATCTGGC		
IL-12	Forward	AAGTAGACTCCAATGGGCAAATG	66	NM_213571.1
IL-12	Reverse	ACGTCTTGCTTGGCTCTTTATAGC		
CXCLi1	Forward	GGCTGGAGCAAAAGGTATGG	58	NM_205018.2
CXCLi1	Reverse	GCACTGGCATCGGAGTTCA		

### Analysis of Antibody Production and Rechallenge With Wild-Type Strain in the *wecB*::Cm-Inoculated Chickens

To assess the level of antibody production in chickens inoculated with the *wecB*::Cm mutant, blood samples were collected from the 5 inoculated chickens at 45 days post-infection. Serum samples were prepared by centrifugation and filtered through a 0.22-μm membrane. All serum samples were stored at −20°C until use. A slide agglutination test using wild-type bacterial antigen preparations was used to detect antibodies in chicken serum that was serially diluted 2-fold, according to the previously reported method (Soria et al., 2015). PBS and serum from uninfected chickens (*n* = 5) were used as a negative control, and anti-O9 serum (Denka, Tokyo, Japan) was used as a positive control. To rechallenge with the wild-type strain in *wecB*::Cm-inoculated chickens (*n* = 5), the chickens were inoculated by oral gavage with a 1.0-ml volume of 10^8^ CFU of the wild-type strain at 35 days after *wecB*::Cm inoculation. The chickens were reared for 14 days and observed twice daily to monitor their clinical signs.

### Statistical Analysis

The bacterial counts were converted logarithmically, and the differences between means of wild-type and *wecB*::Cm mutant obtained for each day were analyzed using Student's *t*-test. For the analysis of cytokine expressions, the statistical comparison was made by one-way ANOVA analysis, followed by Tukey's multiple comparison test to detect differences between uninfected control group, wild-type infected group, and *wecB*::Cm mutant infected group at each day. Both analyses were performed using GraphPad Prism version 9.2.0 (GraphPad Software; San Diego, CA, USA), and *p* < 0.05 was considered statistically significant.

## Results

### Construction and Characteristics of *wecB*-Deficient *S*. Gallinarum Mutant Strain

**Figure 1:**
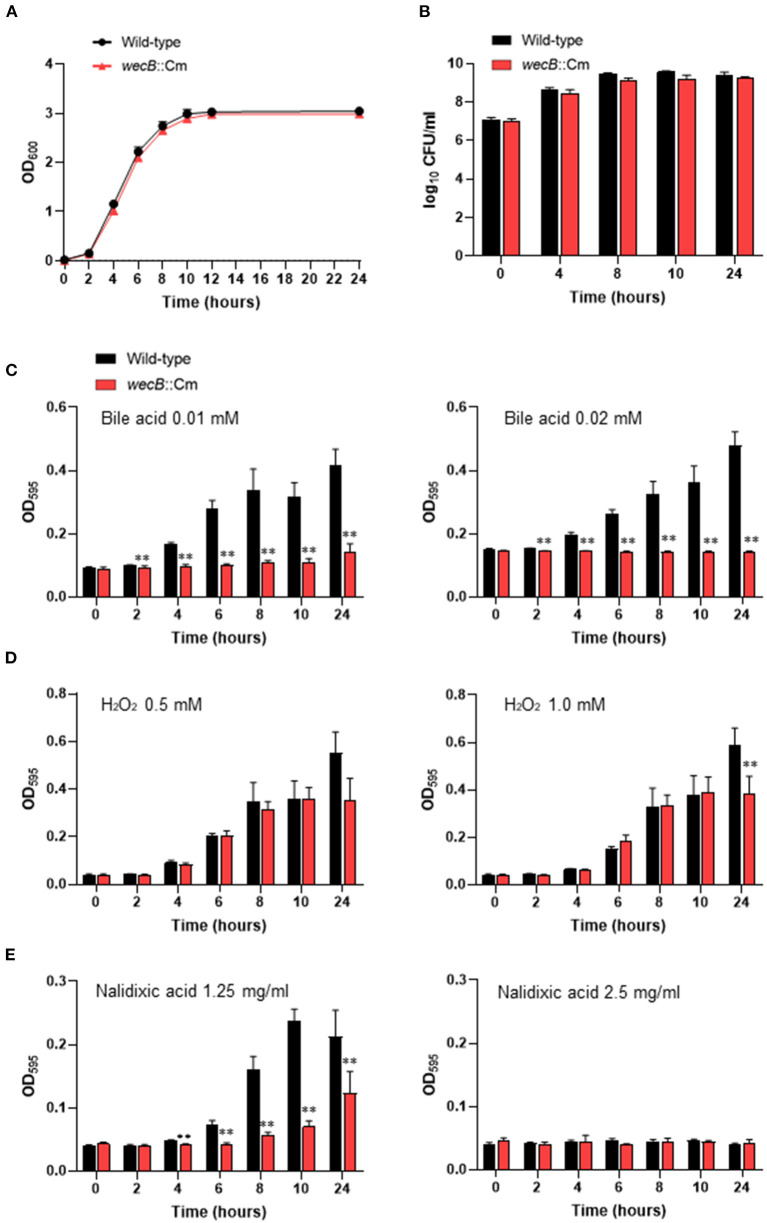
Characteristics of *wecB*::Cm mutant of *S*. Gallinarum. **(A)** Growth curves of wild-type and *wecB*::Cm mutant in Luria-Bertani (LB) broth. The bacteria grown in LB broth for 14 h were diluted to OD_600_ = 1.5 and inoculated 1/100 into the LB broth and cultured at 37°C with shaking (150 rpm). The optical density (OD600) was measured at 0, 2, 4, 6, 8, 10, 12, and 24 h after inoculation. **(B)** Bacterial counts of wild-type and *wecB*::Cm mutant in LB broth. The bacteria were cultivated, as described above. At the indicated time points, 100 μl of each culture was serially diluted in LB broth and 100 μl of each dilution was spread on an LB agar plate. After incubating overnight at 37°C, count the colonies on the plate as colony forming units (CFU). **(C–E)** Bacterial growth characteristics of *wecB*::Cm mutant in LB broth containing bile acid **(C)**, hydrogen peroxide **(D)**, or nalidixic acid **(E)**. The bacteria were grown in LB broth for 14 h and then diluted to 2.0 ×10^7^ CFU/ml. The diluted bacterial culture (50 μl) was mixed with 50 μl of each reagen in 96-well flat-bottom plates and incubated at 37°C without shaking. The optical density (OD_595_) was measured at 0, 2, 4, 6, 8, 10, and 24 h after inoculation. The data are means ± standard deviations based on six wells per group at each time point. The significant differece was shown as **p < 0.01.

We first examined whether the *wecB*::Cm mutant has any alterations in the morphological, physiological, or biochemical properties. The growth curves and viable counts of the mutant in LB broth under aerobic conditions were highly similar to that of the wild-type strain, although the mutant tended to grow slower, the differences were not statistically significant (Figures 1A,B). There were no differences in the morphology and size of the colonies between the mutant and the wild-type strains (data not shown). The effects of the *wecB* gene deletion on the sensitivity of *S*. Gallinarum to bile acids, H_2_O_2_, and nalidixic acid were also evaluated. In the presence of 0.01 mM or 0.02 mM bile acid, the *wecB*::Cm mutant showed significantly lower OD values after 2 h of exposure to bile acid, indicating it was more sensitive than the wild-type strain (Figure 1C). In the presence of 0.5 and 1.0 mM H_2_O_2_, the *wecB*::Cm mutant showed comparable growth to the wild-type strain after exposure for 10 h, although the mutant showed lower OD values at the 24-h time point at 1.0 mM H_2_O_2_ (Figure 1D). To examine whether the *wecB* gene is also relevant for antibiotic resistance, the growth of the *wecB*::Cm mutant was analyzed in LB supplemented with nalidixic acid. At 1.25 mg/ml of nalidixic acid, the growth of the mutant showed a significantly lower OD value than the wild-type strain (Figure 1E). When a high concentration of 2.5 mg/ml was added to the media, both the mutant and wild-type strains abrogated growth.

### Virulence Analysis of the *wecB*-Deficient Mutant in the Infected Chickens

**Figure 2:**
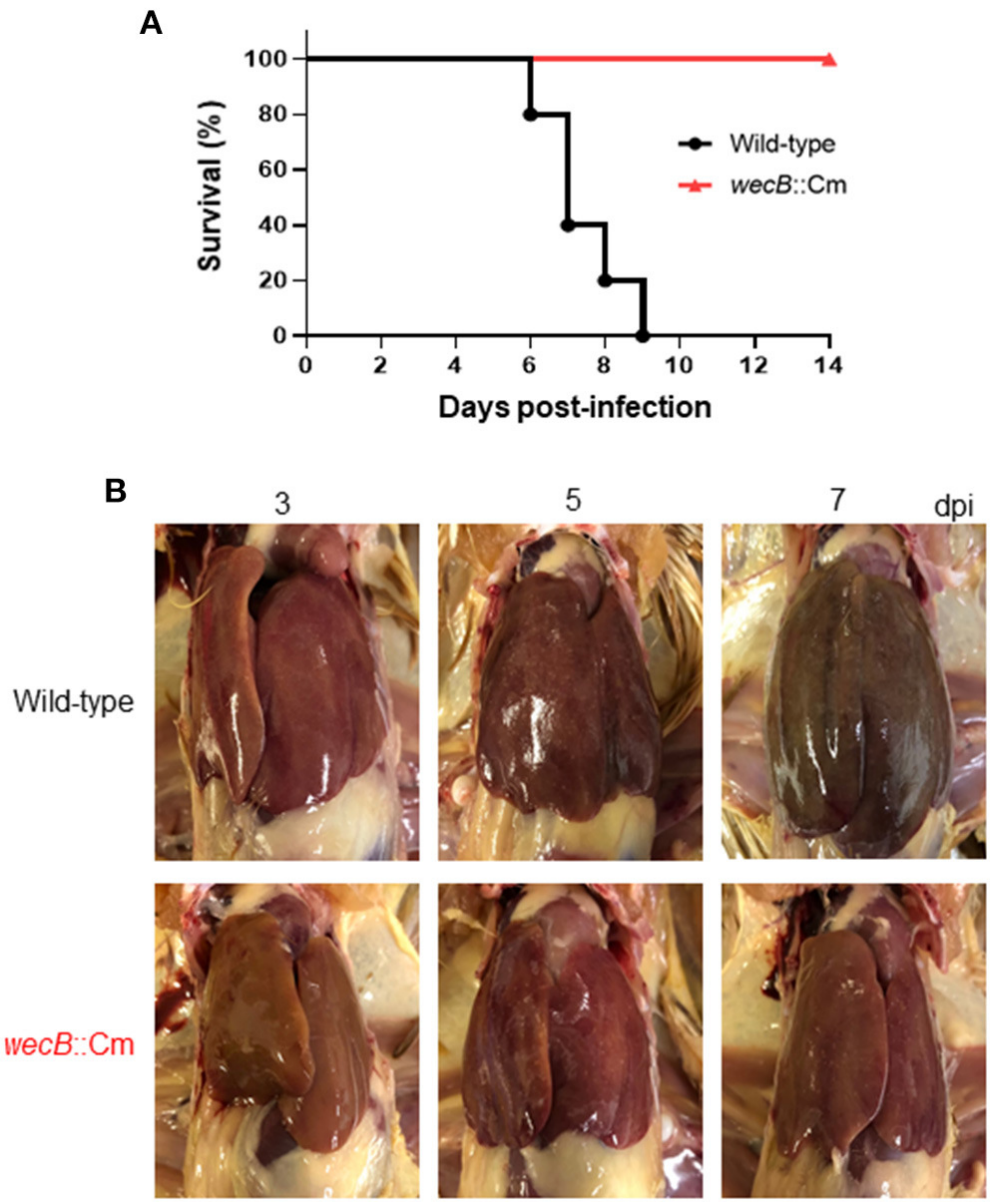
Pathological finding in the chickens orally infected with wild-type or *wecB*::Cm mutant strains. **(A)** Survival curve of chickens orally inoculated with wild-type or *wecB*::Cm mutant. Chickens were orally inoculated with 10^8^ CFU of wild-type or *wecB*::Cm mutant, and survival of the chickens was recorded for 14 days post-infection. **(B)** Gross lesions in the liver of chickens orally inoculated with wild-type or *wecB*::Cm mutant. The livers of pathological changes were observed on 3, 5, and 7 days post-infection (dpi). White lesions and small necrotic foci were observed in wild-type infected chickens on 3 days after infection, and liver hypertrophy and congestion were observed on 7 days after infection. In the chickens infected with *wecB*::Cm mutant, although slight white lesions and small necrotic foci were observed on 5 days after infection, no hypertrophy or swelling was observed.

To investigate the pathogenic roles of the *wecB* gene in the infected chicken *in vivo*, we next analyzed the clinical changes and mortality in chickens orally infected with the *wecB*::Cm mutant or the wild-type *S*. Gallinarum. Chickens infected with 10^8^ CFU of wild-type *S*. Gallinarum showed significant clinical symptoms of fowl typhoid, such as feather disturbance and depression. All chickens infected with the wild-type *S*. Gallinarum died between 6 and 9 days post-infection (Figure 2A). In contrast, the chickens infected with the *wecB*-deficient mutant at the same dose as the wild-type strain exhibited no significant clinical changes and no deaths occurred. They showed a survival rate of 100% even when they were observed up to 25 days post-infection (data not shown).

A previous study has reported that the most characteristic pathological lesions of fowl typhoid, such as hypertrophy, white lesions, and small necrotic foci, were observed in the liver of chickens (Ojima et al., 2021). In this study, we compared the pathological changes in the liver of chickens infected with wild-type and *wecB*::Cm mutant strains. In wild-type infected chickens, white lesions and small necrotic foci were observed on 3 days after infection, and liver hypertrophy and congestion were observed on 7 days after infection (Figure 2B). In contrast, although slight white lesions and small necrotic foci were observed on 5 days after infection, no hypertrophy or swelling was observed in the liver of chickens infected with the *wecB*::Cm mutant.

### Bacterial Colonization of the *wecB*-Deficient Mutant in the Infected Chickens

**Figure 3:**
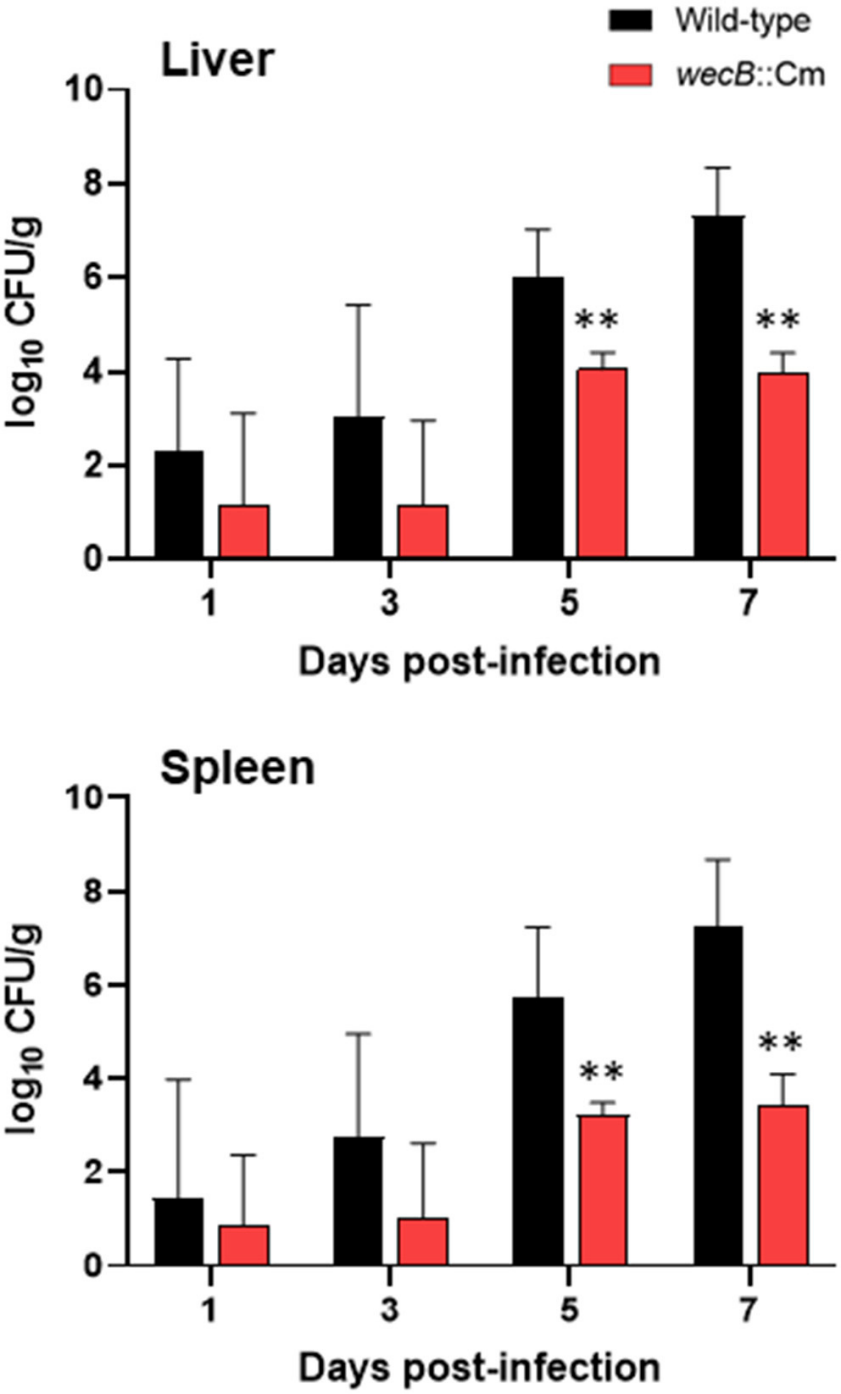
Viable bacterial counts in the liver and spleen of chickens orally inoculated with wild-type or *wecB*::Cm mutant. Chickens were orally inoculated with 10^8^ CFU of wild-type or *wecB*::Cm mutant. The bacteria in the liver and spleen were determined on 1, 3, 5, and 7 days post-infection. The data are means ± standard deviations based on three to six chickens per group at each time point. The significant differences were shown as **p < 0.01.

To investigate the spreading and proliferation abilities of the *wecB-*deficient mutant in the infected chickens, we detected the bacterial burdens in the liver and spleen of chickens at 1–7 days after oral infection with 10^8^ CFU of wild-type or *wecB*::Cm mutant. The results showed that the bacterial counts of wild-type strain in the liver and spleen increased from 1 to 7 days post infection and showed bacterial numbers from 10^2^ CFU/g increasing to 10^8^ CFU/g, respectively, indicating that the wild-type strain rapidly spread to the systemic sites through the gastrointestinal tract and rapidly proliferated in large amounts in the liver and spleen that further caused systemic infection (Figure 3). In contrast, the bacterial counts of the *wecB*::Cm mutant in the liver and spleen slowly increased from days 5 and 7 post-infection and the bacteria numbers remained at 10^4^ CFU/g. There were significantly lower bacterial numbers of the *wecB*::Cm mutant in the liver and spleen than those of the wild-type strain at 5 and 7 days post-infection (*p* < 0.01; Figure 3).

### Pathological Finding and Histological Changes in the Chickens Infected With *wecB*-Deficient Mutant

**Figure 4:**
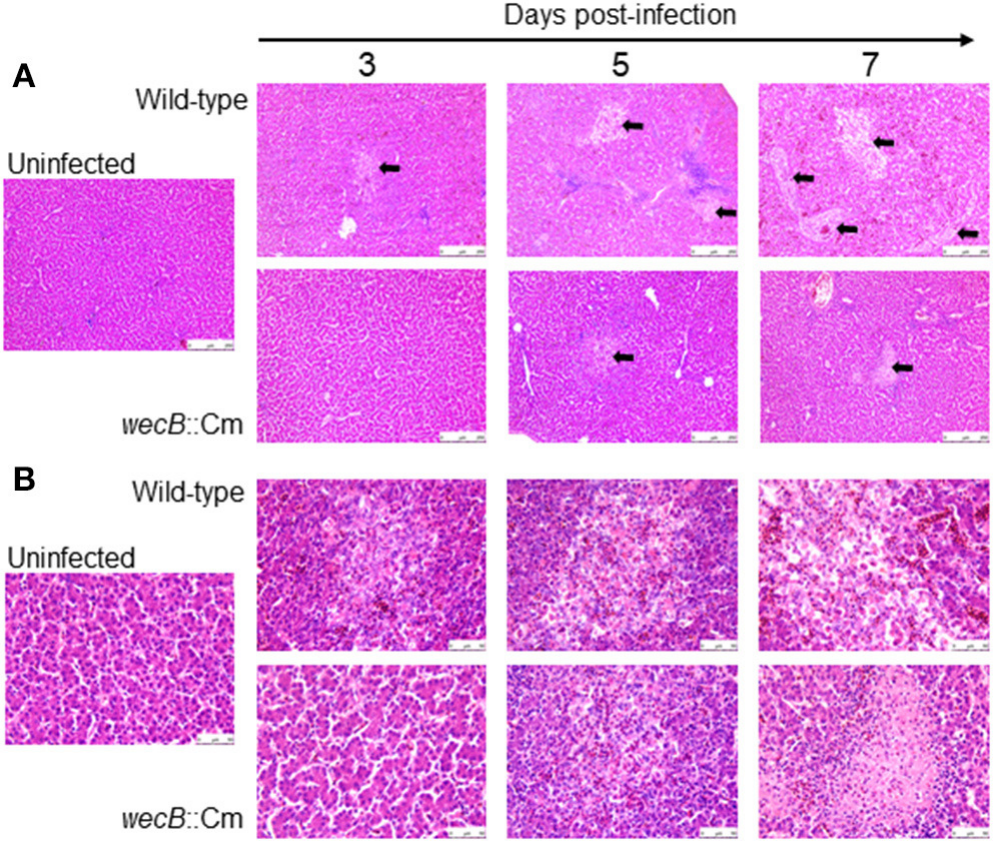
Histopathological changes and microscopic lesions in the livers of chickens orally infected with wild-type or *wecB*::Cm mutant strain. Chickens were orally inoculated with 10^8^ CFU of wild-type or *wecB*::Cm mutant and the livers were collected on 3, 5, and 7 days post-infection. The organs of uninfected chickens were used as the controls. Paraffin sections of the organs were prepared and stained with hematoxylin-eosin (HE). **(A)** Magnification 100 × and **(B)** magnification 400 × . Arrows show lesions which were characterized by marked infiltration of heterophils and lymphocytes with degeneration and necrosis.

**Figure 5:**
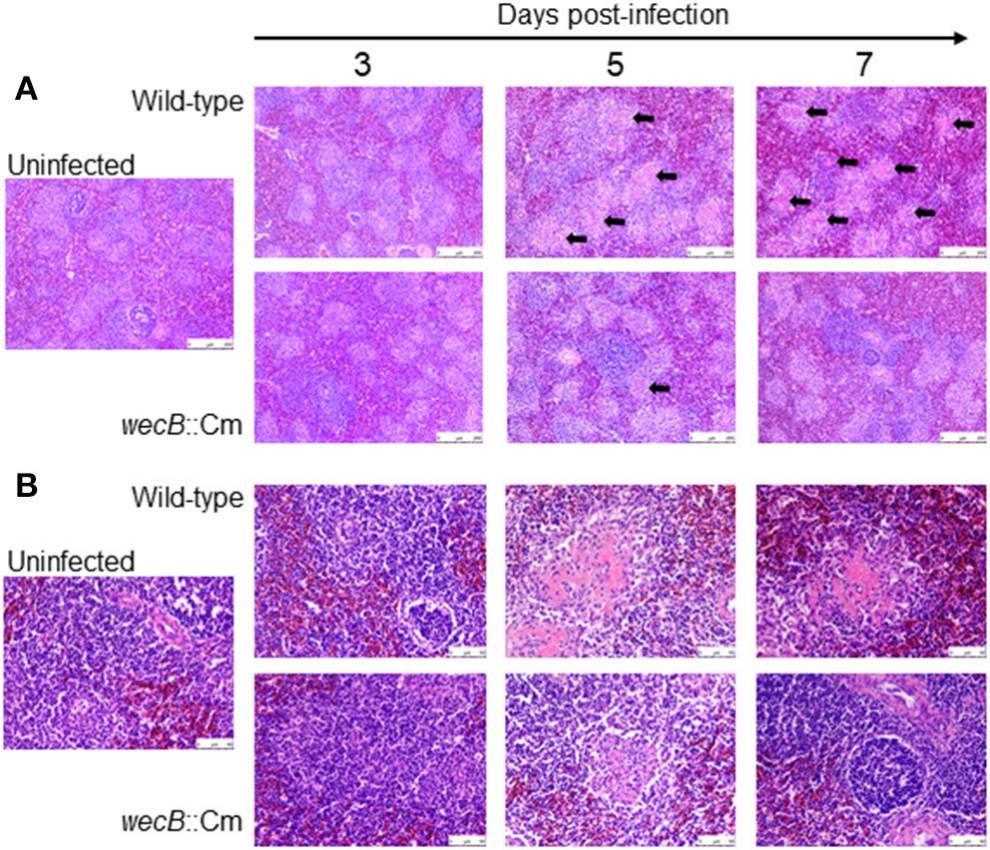
Histopathological changes and microscopic lesions in the spleens of chickens orally infected with wild-type or *wecB*::Cm mutant strain. Chickens were orally inoculated with 10^8^ CFU of wild-type or *wecB*::Cm mutant and the livers were collected on 3, 5, and 7 days post-infection. The organs of uninfected chickens were used as the controls. Paraffin sections of the organs were prepared and stained with HE. **(A)** Magnification 100 × and **(B)** magnification 400 × . Arrows show degeneration and necrosis in white pulp.

To understand whether the *wecB* gene is related to tissue inflammation caused by *S*. Gallinarum, we performed histopathological examinations on the liver and spleen of infected chickens. In the livers, lesions became detectable on 3 days after infection and the lesions were characterized by marked infiltration of heterophils and lymphocytes with degeneration and necrosis of hepatocytes (Figure 4). In contrast, very limited or no significant pathological changes were observed in the liver of *wecB*::Cm mutant-infected chickens. In the spleen, histopathological changes, such as degeneration and necrosis in white pulp, were observed on 5 and 7 days post-infection in the wild-type infected chickens (Figure 5). In contrast, although some mild white pulp necrosis was observed in chickens infected with the *wecB*::Cm mutant on 5 days after infection, no lesions were observed on 3 and 7 days post-infection.

### Immune Responses in the Chickens Infected With *wecB*-Deficient Mutant

**Figure 6:**
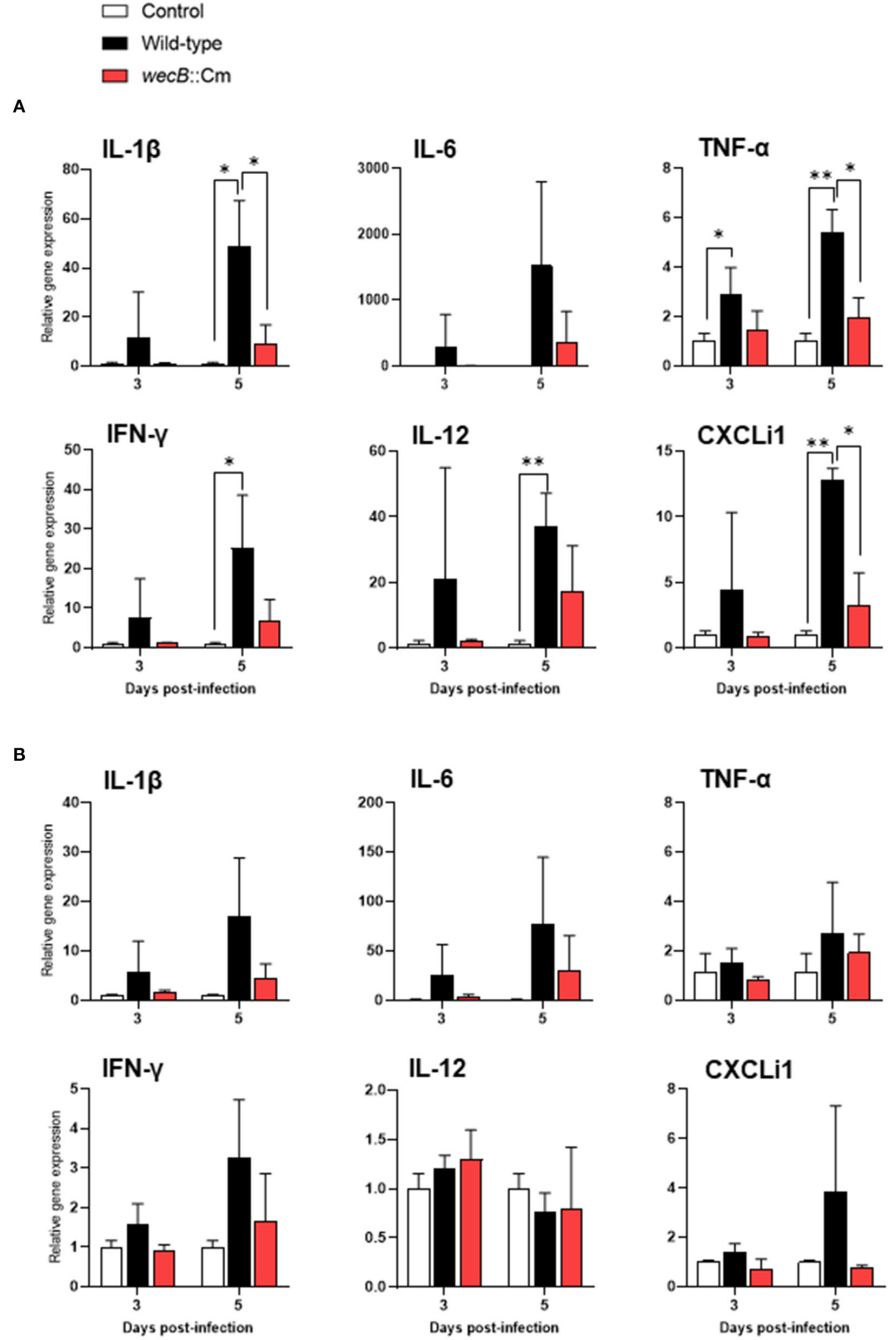
Expression of cytokine and chemokine in the liver **(A)** and spleen **(B)** of chickens infected with wild-type or *wecB*::Cm mutant strains. Chickens were inoculated orally with 10^8^ CFU of wild-type or *wecB*::Cm mutant. The liver and spleen of the chickens were collected on 3 and 5 days post-infection, and the expression of interleukin-1β, IL-6, tumor necrosis factor-α, interferon-γ, IL-12, and CXCLi1 was determined by quantitative RT-PCR. Data were expressed as means ± standard deviations of fold changes in gene expression of the organs from infected groups relative to those from the uninfected control group (three chickens per group at each time point). Statistical analysis was performed using one-way ANOVA analysis followed by Tukey's multiple comparison test to compare infected chickens with uninfected controls. The significant differences were shown as *p < 0.05, **p < 0.01.

To further analyze the immune responses in the chickens infected with *wecB*::Cm mutant, we determined the expression of selected cytokine and chemokine genes of IL-1β, IL-6, TNF-α, IFN-γ, IL-12, and CXCLi1 in the liver and spleen of chickens after infection with wild-type or *wecB*::Cm mutant strains, respectively. Results showed that the expression of TNF-α was significantly induced in the liver of chickens infected with the wild-type strain at 3 and 5 days post-infection. The expressions of IL-1β, IL-6, TNF-α, IFN-γ, IL-12, and CXCLi1 were also markedly increased in the livers of wild-type infected chickens on 5 days post-infection. In contrast, the expression of IL-1β, TNF-α, and CXCLi1 in the livers of chickens infected with *wecB*::Cm mutant was significantly lower than that of the chickens infected with the wild-type strain (Figure 6A). The expression levels of the cytokines were almost the same as those of the uninfected control chickens. In the spleen, neither wild-type nor *wecB*::Cm mutant infections significantly expressed any tested pro-inflammatory cytokine and chemokine genes. There were no significant differences between the chickens infected with wild-type and *wecB*::Cm mutant strains (Figure 6B).

### Persistent Colonization by Bacteria and Antibody Production in the Chickens Infected With *wecB*::Cm Mutant

**Figure 7:**
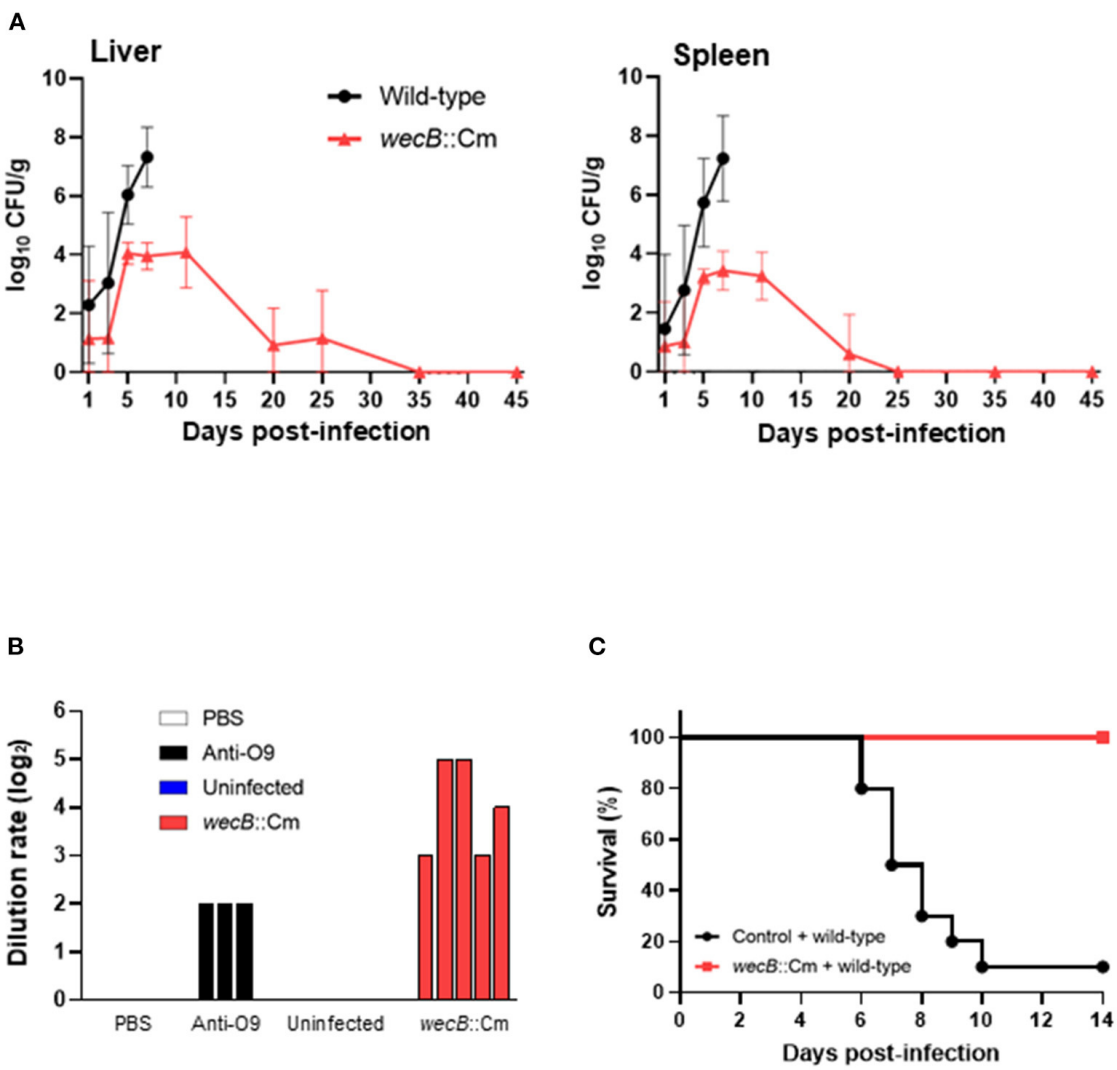
Kinetics of bacterial colonization and protective immune responses in the chickens orally infected with *wecB*::Cm mutant. **(A)** Viable bacterial numbers in the liver and spleen of chickens infected with wild-type or *wecB*::Cm mutant strains. Chickens were orally inoculated with 10^8^ CFU of wild-type or *wecB*::Cm mutant, and the number of bacteria in the organs was determined on 1, 3, 5, 7, 11, 20, 25, 35, and 45 days post-infection. The data are means ± standard deviations based on three to six chickens per group at each time point. **(B)** Antibody production in chickens infected with *wecB*::Cm mutant strain. Five Chickens were orally inoculated with 10^8^ CFU of *wecB*::Cm mutant, and the anti-*S*. *gallinarum* antibodies were determined by serum agglutination test on 45 days after infection. PBS and serum from uninfected chickens (*n* = 5) were used as a negative control, and anti-O9 serum was used as a positive control. **(C)** Survival curve of chickens orally inoculated with *wecB*::Cm mutant and re-challenged with wild-type strain on 35 days after initial inoculation. *wecB*::Cm-inoculated chickens were re-challenged with 10^8^ CFU of the wild-type strain, and the survival of chickens was recorded for 14 days after re-infection with the wild-type strain.

To determine whether the loss of virulence was due to the inability of *wecB*::Cm mutant to grow in the host, the numbers of bacteria in the livers and spleens of chickens infected with the mutant were monitored for up to 45 days post-infection (Figure 7A). On 5 days post-infection, a few bacteria, ~2 ×10^4^ CFU/g in the livers and 1 ×10^3^ CFU/g in the spleens, were recovered from the chickens infected with the *wecB*::Cm mutant compared with the wild-type strain. Starting from the 5th day after infection and continuing to 25 days after infection, *wecB*::Cm bacteria were reduced and eliminated from the chickens, and no bacteria were recovered from the organs 35 days after infection. In addition, we further determined whether *wecB*::Cm-inoculated chickens induced anti-*S*. Gallinarum antibody production. The results showed that all the chickens significantly produced antibodies compared with the uninfected controls (Figure 7B). Furthermore, the chickens with high antibodies and rechallenged with 10^8^ CFU of wild-type *S*. Gallinarum showed significantly higher survival rates than the control chickens that were not initially inoculated with the *wecB*::Cm mutant and did not produce antibodies against *S*. Gallinarum (Figure 7C).

## Discussion

*S*. Gallinarum is a natural aflagellate and poultry-specific *Salmonella* that causes severe systemic infection affecting domestic fowl typhoid and leading to high mortality (Shivaprasad and Barrow, 2008; Foley et al., 2013; Huang et al., 2019, 2020). However, less is known about the pathogenic characteristics of *S*. Gallinarum and the pathogenic mechanism of systemic infection in chickens. We recently established an oral infection model and investigated the pathogenic characteristics and dynamic process of *S*. Gallinarum-induced systemic infection *in vivo*, mimicking the natural infection in chickens (Ojima et al., 2021). In this study, to reveal the pathogenic mechanism and understand the biological function of ECA of *S*. Gallinarum for systemic infection in chicken, we constructed a *wecB*-deficient (ECA-negative) mutant of *S*. Gallinarum and evaluated the mutant strain for its virulence in an oral infection model of chickens. Our results demonstrate that the *wecB* deleted mutant is sensitive to bile salts, deoxycholic acid, and nalidixic acid, and the resulting strain was significantly attenuated *in vivo* infection of chickens. Interestingly, *wecB* deleted mutant can persist in chicken organs at low levels for up to 25 days post-infection and can induce protective immune responses, indicating that it is potentially possible to use the *wecB* mutant, an ECA-negative strain of *S*. Gallinarum, as a live-attenuated vaccine strain for controlling fowl typhoid.

The *wecB* gene encodes UDP-*N*-acetylglucosamine-1-phosphate transferase that contributes to the construction of ECA_PG_ (Rai and Mitchell, 2020). The function of ECA in *Enterobacteriaceae* species has been mainly studied in the bacteria that have a wide range of hosts, such as *E. coli, S*. Typhimurium, and *S*. Enteritidis (Gilbreath et al., 2012; Jaiswal et al., 2015; Jiang et al., 2020; Rai et al., 2021). ECA consists of trisaccharide repeating unit, GlcNAc, ManNAcA, and 4-acetamido-4,6-dideoxy-D-galactose (Fuc4NAc). The genes necessary for ECA synthesis are located within the *wec* operon, including the *wecB* gene (Blattner et al., 1997; Whitfield et al., 1997; Rai and Mitchell, 2020). The product of the *wecB* gene is a homodimeric enzyme, UDP-*N*-acetylglucosamine 2-epimerase, that is responsible for synthesizing UDP-ManNAcA from UDP-GlcNAc, reversibly epimerizes at carbon position 2 between UDP-GlcNAc and UDP-*N*-acetylmannosamine (Rick et al., 1985; Meier-Dieter et al., 1990). Studies on ECA function have shown that it plays a vital role in bacterial physiology and interaction with the environment and hosts. Previous studies have reported that ECA is linked to virulence in several species of bacteria and the pathogenic function of ECA seems to differ in each species (Barua et al., 2002; Tamae et al., 2008; Nichols et al., 2011; Mitchell et al., 2018). ECA production in *Serratia marcescens* is linked to flagellar assembly and swarming motility (Castelli et al., 2008). Several studies have reported that ECA plays a role in the virulence of *S*. Typhimurium in mice model of infection (Gilbreath et al., 2012; Bridge et al., 2015; Liu et al., 2020; Rai and Mitchell, 2020). Although ECA is present in many species, each species has evolved a unique way to utilize ECA or ECA biosynthesis in a manner that is most conducive to survival (Rai and Mitchell, 2020). Compared with the functional studies of ECA in *E. coli, S*. Typhimurium, and other Gram-negative bacteria, less is known about the function and pathogenic effect of ECA in the bird-specific bacterium, *S*. Gallinarum, that causes systemic infection in chickens. This study showed that *wecB* deletion strains of *S*. Gallinarum have no significant changes in the growth kinetics and colony morphology *in vitro*, but the sensitivity to bile acid and nalidixic acid was significantly higher than those of the wild-type strain (Figure 1). Importantly, the pathogenicity of the *wecB* deletion mutant was severely attenuated during the oral infection (Figure 2A). The mutant strain did not kill the chickens, and the number of bacteria in the organs of the *wecB* mutant infected chickens was significantly less than that of the chickens infected with the wild-type *S*. Gallinarum (Figure 3). Furthermore, the production of pro-inflammatory cytokines in the liver induced by the *wecB* mutant was significantly lower than that of the wild-type infected chickens (Figure 6A). However, there were no significant changes in the production of the pro-inflammatory cytokines in the spleen infected with wild-type or *wecB* mutant compared with uninfected control (Figure 6B). These results suggest that the immune response to *S*. Gallinarum infection may be more sensitive in the liver than in the spleen, although wild-type infected chickens had nearly equal numbers of bacteria in the liver and spleen. Definitive *in vivo* studies on the effects of ECA of *Salmonella* are very limited and the precise mechanism of attenuation remains unclear. Previous studies have reported that ECA of *S*. Typhimurium is related to bile resistance, and the bile sensitivity may be responsible for the oral virulence defect of the *wecA* mutant strains, which is consistent with our study on *wecB* mutant of *S*. Gallinarum (Ramos-Morales et al., 2003; Gilbreath et al., 2012). Molecules expressed on the surface of bacterial cells have been shown to act as pathogen-associated molecular patterns and are known to act as ligands for immune signal receptors (Castelli and Véscovi, 2011; Jorgenson et al., 2016; Rojas et al., 2018). The lack of ECA on the surface of the *wecB* mutant may change the initial steps of the host's immune response activation and can make it impossible to clear the organism from the systemic sites of the chickens. Although the virulence of the complementary *wecB* mutant has not been tested, we expect that the complementary *wecB* mutant may exhibit bile acid resistance and restore its pathogenicity in chicken infection like the wild-type strain. Future studies should include the construction of complementary *wecB* mutant and focus on the characterization of the immune response of chicken to the ECA-negative mutant strain of *S*. Gallinarum.

Unlike *S*. Enteritidis and *S*. Typhimurium, *S*. Gallinarum induces a severe, septicaemic, and systemic infectious disease in poultry, rather than gastrointestinal infections (Wigley, 2017; Huang et al., 2019; Ojima et al., 2021). A key component of the pathogenesis of *S*. Gallinarum in the chicken systemic infection model by oral inoculation is the ability of the bacteria to spread and colonize systemic sites. During the oral infection, the process requires bacteria to effectively cross the intestinal barrier, establish and survive in the intracellular niche, and then spread to the peripheral sites. Our results showed that the *wecB* mutant strain of *S*. Gallinarum was able to persistently colonize the systemic sites of chickens, even up to over 25 days post-infection with a low level of bacterial numbers in the liver and spleen (Figure 7A), suggesting that the attenuation of *wecB* mutant may not be due to the inability to spread in the host adequately, but may reflect the inability of ECA mutant to obtain the high number of bacteria that cause severe pathological changes in the host (Figure 3). Previous studies have shown that mutation of some genes, such as *aroA, purE*, or *wecA*, in *S*. Typhimurium leads to establish the persistent infection of mice *in vivo* (Bohm et al., 2018; Mitchell et al., 2018; Rai et al., 2021). The persistent bacteria within the organs could result in increased or prolonged immune stimulation within the host (Gilbreath et al., 2012; Tang et al., 2018; Liu et al., 2020). Given these findings, the gene mutant strains have been considered potential live-attenuated vaccine candidates (Huang et al., 2016; Foster et al., 2021). Maintaining a delicate balance between attenuated virulence and optimal immunogenicity is a major consideration for the future development of live-attenuated vaccine strains (Gilbreath et al., 2012). Our results clearly show that the *wecB* mutant strain of *S*. Gallinarum is significantly attenuated in the chicken systemic infection model without significant pathogenicity and low inflammatory cytokine induction. It can establish persistent colonization in organs at a low level for up to 25 days post-infection and can induce a protective immune response in the inoculated chickens. Therefore, the results presented here suggest that *wecB* mutant may serve as an excellent viable attenuated vaccine strain to protect against *S*. Gallinarum infection in chickens.

## Data Availability Statement

The datasets presented in this study can be found in online repositories. The names of the repository/repositories and accession number(s) can be found in the article/supplementary material.

## Ethics Statement

The animal study was reviewed and approved by the Institutional Animal Care and Use Committee of Kitasato University. Written informed consent was obtained from the owners for the participation of their animals in this study.

## Author Contributions

D-LH and HO: conceptualization. SO, HO, RO, XY, MS, MO, and D-LH: methodology. SO, RO, MS, and HO: investigation. SO and RO: data analysis and curation. SO: writing—original draft preparation. D-LH, HO, KY, TH, and MO: writing—review and editing. SO, HO, and D-LH: visualization. D-LH: supervision, project administration, and funding acquisition. All authors have read and agreed to the published version of the manuscript.

## Conflict of Interest

The authors declare that the research was conducted in the absence of any commercial or financial relationships that could be construed as a potential conflict of interest.

## Publisher's Note

All claims expressed in this article are solely those of the authors and do not necessarily represent those of their affiliated organizations, or those of the publisher, the editors and the reviewers. Any product that may be evaluated in this article, or claim that may be made by its manufacturer, is not guaranteed or endorsed by the publisher.
